# Methodology for Sound Quality Analysis of Motors for Automotive Interior Parts through Subjective Evaluation

**DOI:** 10.3390/s22186898

**Published:** 2022-09-13

**Authors:** Sung-Yuk Kim, Sang-Chul Ryu, Yong-Du Jun, Young-Choon Kim, Jong-Seok Oh

**Affiliations:** 1 Future Automotive Intelligent Electronics Core Technology, Kongju National University, Cheonan 31080, Korea; 2 Department of Mechanical Engineering, Kongju National University, Cheonan 31080, Korea; 3 Division of Mechanical & Automotive Engineering, Kongju National University, Cheonan 31080, Korea; 4 Department of Intelligent Mobility Engineering, Kongju National University, Cheonan 31080, Korea; 5 Department of Future Automotive Engineering, Kongju National University, Cheonan 31080, Korea

**Keywords:** sound quality, sensibility, automotive, motor, interior parts

## Abstract

With the development of autonomous vehicles, activities in the indoor spaces of autonomous vehicles are diversifying. Therefore, as the operating range of the interior parts increases, the occupant becomes sensitive to the operating noise of autonomous vehicles. Therefore, to reduce operating noise, it is necessary to analyze the causal relationship between the mechanical/electrical noise characteristics of the motor and sound quality. In this paper, we propose a methodology to analyze the relationship between the noise frequency components and the sound quality of small motors used in automobile interior parts. Two types of motors were selected for this study, and noise measurements and analyses were performed by applying the design proposed in this study. Subjective sound quality evaluations were conducted using the 12 pairs of adjectives extracted from the survey. The results suggest that subjective sound quality evaluation scores should be converted to Z-scores to ensure the reliability of the statistical analysis. In addition, we present a critical sound quality value that can be used as a criterion for determining whether the sound quality is positive (good quality) or negative (bad quality). Sound quality regression models explain the causal relationship between rotational frequency components of the motor and subjective sound quality characteristics. Thus, a method for analyzing the effect of the rotational frequency component of the motor on the sound quality is presented, which suggests that it can be used as basic research data to improve the noise performance of the motor.

## 1. Introduction

Recently, the noise generated by automobiles has been significantly reduced owing to the advent of eco-friendly automobiles, such as hybrid, electric, and fuel cell vehicles. However, with the improvement of indoor quietness and quality of sensibility, noise generated from the driving mechanism of interior parts, such as power windows, panoramic sunroofs, and power seats, which were previously not an issue, is relatively highlighted. In particular, as the power seat for automobiles is being developed as a seat system with multiple functions (e.g., swivel, belt in seat, long rail slide, and wireless power transmission) as the level of autonomous vehicles develops along with electric vehicles [1,2,3,4], improvement of the noise of the driving mechanism in the interior is gaining significance [5].

The mechanisms of automobile interior parts, such as power seats, are generally composed of motors, shafts, gears, rails, and frame structures, which are vulnerable to noise and vibration because the mechanisms are intensively arranged in a narrow space [6,7,8]. In particular, in autonomous vehicles, because the seat slide rail has a long rail structure with a length of 1000 mm or more, the stiffness of the rail is reduced and the driving speed is increased, resulting in an overall increase in driving noise [9]. In addition, because the noise is generated at the closest distance to the driver or passengers, the seat slide rail reacts sensitively even to low-level noise. Therefore, there is a need for an analysis method to improve the level of sound quality and reduce the noise of the seat-driving mechanism.

The analysis of sound quality in the automobile field is divided into objective and subjective evaluation methods. First, in an objective evaluation, the sound pressure level and psychoacoustic parameters are the most representative methods. The sound pressure level is a physical quantity representing the magnitude of sound pressure, and A, B, C, D, G, and Z weight filters can be applied. Among them, the A-weighted decibel, which reflects the equal-loudness contour, is widely used not only in automobiles but also in general home appliances. The sound pressure level can be analyzed in the frequency domain using FFT and waveform analysis in the time domain; however, it is difficult to represent sound quality because it represents the magnitude of the sound. In the case of psychoacoustic parameters, Loudness, Sharpness, Roughness, Fluctuation strength, and Tonality are sound quality factors mainly used in the automotive field, and they are determined by applying a weight to a specific frequency band or the influence of the modulation frequency [10,11,12]. These are mainly advantageous for quantifying and evaluating overall sound quality characteristics of noise. However, when improvement of sound quality or a specific tone is required, there is a disadvantage in analysis because it is necessary to improve such as design change by understanding the influence of noise sources on sound quality rather than evaluating sound quality. Rather than evaluating sound quality using only psychoacoustic parameters, most of the subjective auditory evaluations are conducted together to verify the correlation with psychoacoustic parameters. This is because, depending on the product, there are cases in which the data trends and hearing sensations of psychoacoustic parameters are different. If psychoacoustic parameters do not follow the characteristics of hearing, new parameters are created by correcting parameter constants or combining parameters such as psychoacoustic annoyance or sensory pleasantness. The models of psychoacoustic annoyance and sensory pleasantness are presented in Equations (1) and (2).

(1) $$PA=N_{5}\left(1+\sqrt{w_{S}^{2}+w_{\mathrm{FR}}^{2}}\right),$$

(2) $$\frac{P}{P_{0}}=e^{-{\left(0.023\frac{N}{N_{0}}\right)}^{2}}e^{-1.08{\left(\frac{S}{S_{0}}\right)}^{2}}e^{-0.7\frac{R}{R_{0}}}\left(1.24-e^{-2.43\frac{T}{T_{0}}}\right),$$

where *PA* is psychoacoustic annoyance, and *P* is sensory pleasantness. *N* is Loudness (sone), *S* is Sharpness (acum), *R* is Roughness (asper), *F* is Fluctuation strength (vacil), *T* is tonality (tu), *N*_5_ is percentile loudness, *w*_S_ is the effect of *S*, *w*_FR_ is the influence of *F* and *R*, and 0 represents the relative value that the sensory pleasantness of the sound under investigation is being compared to [13,14]. In order to utilize psychoacoustic parameters such as *PA* and *P*, significant time and effort are required because it is necessary to verify the significant correlation between the loudness, sharpness, roughness, and fluctuation strength data of the target product and the sense of hearing. Therefore, there is a need for an objective evaluation method that can be used more quickly and simply in the initial development stage of products.

Next, for sound quality analysis through subjective evaluation, it is necessary to select the method that best represents the multidimensional psychological state of human beings. In general, the semantic differentiation method is the most commonly used method. It is an evaluation method based on the scales of the subjects by presenting various pairs of opposite adjectives [12,15,16]. It is suitable for the evaluation and analysis of multidimensional objects, such as sound. However, to utilize the semantic differential method, it is important to select an adjective suitable for evaluation. In the automobile field, subjective evaluation using the semantic differential method has mainly been conducted on exterior and chassis parts, such as engines, tires, doors, and transmissions, and the adjective vocabulary system for evaluating them has been well established [17,18,19,20,21,22]. However, in the case of automobile interior parts, research on sound quality remains limited. In particular, because a rotating body such as a motor generates noise simultaneously in both the electrical and the structural domains, the sound quality characteristics are different from those of general mechanical mechanisms. Therefore, it is important to understand the effect of each noise source on the sound quality because it has not yet been studied.

In this study, a methodology to analyze the effects of the mechanical and electrical noise characteristics of small motors used in automobile interior parts on sound quality is proposed. First, two types of motors were selected for the study, and noise measurements were performed. Next, 12 pairs of sound quality adjectives were derived by conducting a survey to select the adjectives to be used for subjective sound quality evaluation. Third, a subjective sound quality evaluation was performed using the measured motor noise and sound quality adjectives, and a regression model was derived using the evaluation results. The correlation between the noise source characteristics and the sound quality was analyzed by deriving the sound quality critical value of the motor using the regression model. Lastly, the proposed method was verified by comparative analysis of the sound quality predictive accuracy of the presented sound quality model and psychoacoustic models. The sound quality analysis methodology presented in this study is simpler than sound quality analysis using psychoacoustic parameters because it uses only sound pressure levels. In addition, the accuracy of sound quality analysis is high; hence, it can be used in the initial development stage of the product.

## 2. Measurement of Operating Noise of DC Motor

### 2.1. Excitation Force of DC Motor

Acoustic noise generated by DC motors is generally classified into mechanical noise and electrical noise. First of all, mechanical noise is greatest at the brush switching frequency generated by the contact between the commutator and the brush. The Equation for deriving the frequency component for this is as follows:

(3) $$f_{B}=mLCM_{B}f_{f},$$

where *m* is a positive integer, *LCM*_B_ is the least common multiple of the number of commutator slots and brushes, and *f*_f_ is the basic rotation frequency. The positive integer *m* denotes a harmonic order.

The main causes of low-frequency acoustic noise in motors are unbalance and misalignment. Unbalance can be caused by manufacturing or assembly errors, physical damage, wear, fouling, or loss of balance weights. Others may be due to manufacturing or assembly errors, physical damage, wear, fouling, or loss of balance weights. On the other hand, misalignment is mainly caused by the misalignment of the centerline, but can also be caused by physical movement. The equations for them are as follows:

(4) $$f_{U}=mf_{f},$$

(5) $$f_{M}=2mf_{f},$$

where *f*_U_ is the unbalance frequency, and *f*_M_ is the misalignment frequency.

Next, as electrical acoustic noise, torque ripple generated by the electromagnetic force of the motor is the main noise source. The Equation is as follows:

(6) $$f_{E}=mLCM_{E}f_{f},$$

where *LCM*_E_ is the least common multiple of the number of commutator slots and the number of magnet poles.

### 2.2. Measurement

**Figure 1:**
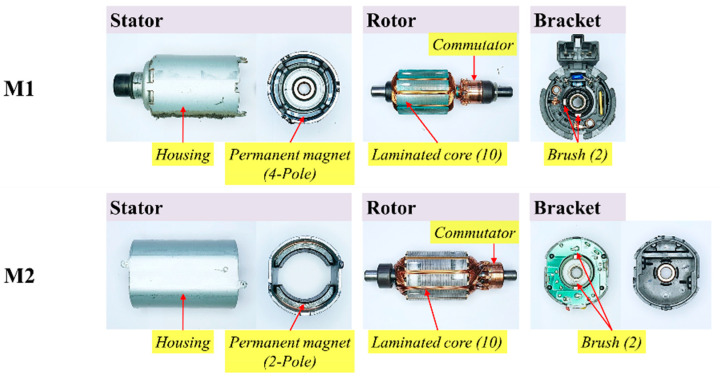
DC motor samples.

The main purpose of the motor dynamometer experiment was to acquire sound sources to be used for noise source identification and subjective sound quality evaluation. Figure 1 shows the motor used in this experiment. Among the various DC motors currently used for common power seats, two types used for the slide mechanism were selected. First, the experimental factors of the DC motor were selected as the direction of rotation, the number of magnet poles, and the load torque. The rotation direction was clockwise and counterclockwise, the number of magnet poles was set to two and four poles, and the load torque was set to 0.04 N·m, 0.08 N·m, and 0.12 N·m. In the case of the load torque, the load torque applied to the motor when the power seat slide mechanism is operated is generally 0.1 N·m or less, assuming that the weight of the occupant is about 75 kg. However, considering the case of driving under harsher weight conditions, 0.12 N·m was selected as the maximum.

**Figure 2:**
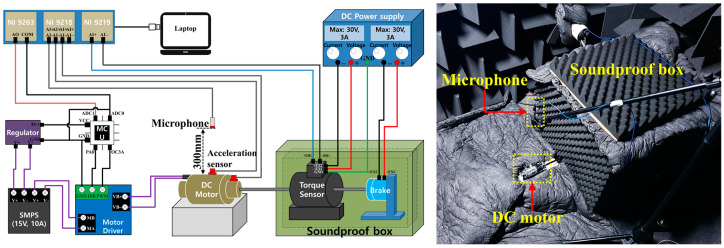
Motor dynamometer experiments.

Figure 2 is a schematic diagram of a motor dynamometer devices that is used in an actual experiment. Noise measurement was performed using a Simcenter SCADAS front-end in a semi-anechoic room where the background noise was kept below 25 dB (A). Table 1 is the design matrix used in this experiment, which applied a full factorial experiment design. The order of experiments was randomized, and all experiments were repeated three times.

## 3. Sound Quality Evaluation

### 3.1. Survey for Extracting Sound Quality Adjectives

Subjective sound quality evaluation is used to investigate human sensibility and psychological conditions regarding sound in various ways. These methods include paired comparison, grade evaluation, and semantic differential methods. The semantic differential method is a measurement scale designed to determine the subjective perception of a person and affective reactions to the properties of concepts, objects, and events by making use of a set of bipolar scales. It can be analyzed at a multidimensional level using pairs of several contrasting adjectives, with seven-point scales being the most common form.

To use the semantic differential method, it is important to select adjectives that can express objects in multiple dimensions. In this section, a survey was conducted on the basis of the adjective vocabulary established from existing automotive and sound quality research, and the sound quality evaluation adjectives of automotive interior parts were established.

#### 3.1.1. Survey Method and Target

**Figure 3:**
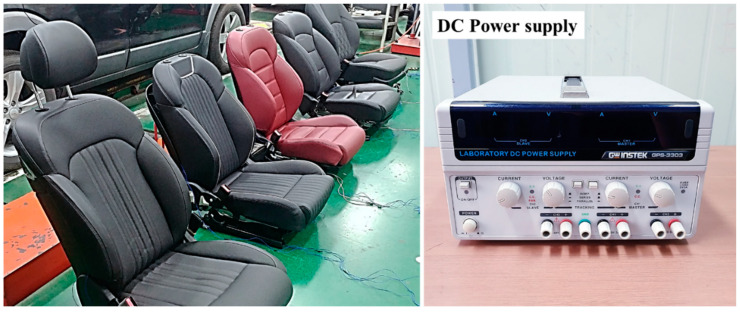
Power seat samples used for sensibility vocabulary survey.

The purpose of the survey was to derive adjectives for evaluating the operating sound quality of motors for automobile interior parts. The overall language of the questionnaire was Korean, and sound quality adjectives were presented in both English and Korean. There were 132 adjectives used in the survey, which were extracted from the literature related to sound quality in the automobile field [17,18,19,20]. Questionnaires filled out with 132 adjectives were distributed to the subjects, and the survey objects were selected as power seats for vehicles operated by small motors. At the time of the survey, an environment that could be directly driven on a vehicle power seat was provided such that the subjects could consider both their past and their present experiences. For the questionnaire, a multi-response method with no limit on the number of adjectives was adopted such that various adjectives could be selected. The subject was allowed to sit freely on the seat and operate on the slide. Because the slide operation noise was at a relatively low level, the head of the subject was brought into contact with the headrest such that the structural noise could be heard more clearly. The seat sample used in the survey was a total of five seats, as shown in Figure 3, all of which were selected as power seats of different car models. A total of 100 undergraduate and graduate students at Kongju National University (84 males and 16 females) aged between 22 and 39 years (median age; 30.5 years) participated in the survey. The survey was conducted in groups of five people, freely sitting on the seat and operating the automobile while filling out the questionnaire. By controlling the exchange of opinions among the subjects regarding questionnaire preparation, judgment errors due to communication were eliminated.

#### 3.1.2. Results of Survey

Table 2 summarizes the results of the frequency analysis of the sound quality adjective survey. In the survey, the adjective that the subjects selected the most was deep, and the adoption rate was approximately 67%, which was less than expected. There were only three adjectives that had an adoption rate greater than 50%: deep, rumbling, and soft. Therefore, the results of the sound quality adjective survey were reanalyzed by applying the percentage to the ranking rather than the frequency ratio.

The percentage criterion for ranking was set as the top 15%. Consequently, a total of 16 adjectives included in the questionnaire were derived: deep, high, rumbling, buzzing, soft, slow, stable, quiet, comfortable, expensive, smooth, monotonous, fluctuating, light, heavy, and weak. The selected adjectives were reorganized into adjective pairs with their respective characteristics and classified as listed in Table 2.

### 3.2. Subjective Sound Quality Evaluation

#### 3.2.1. Evaluation Method

The sound source used for the subjective sound quality evaluation was the motor operating noise recorded in the experiments discussed in Section 2 and listed in Table 1. There were 12 types of sound sources, and the same sound source was duplicated three times such that the subject listened to a total of 36 sound sources. The playback of the sound source proceeded in random order.

**Figure 4:**
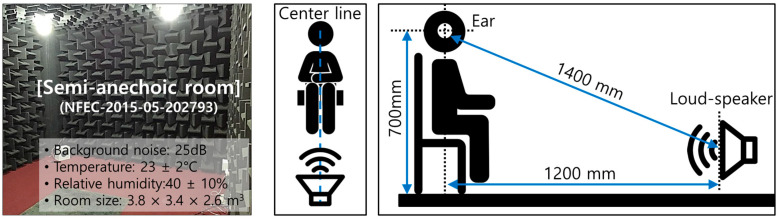
Loud-speaker and environmental conditions for sound quality evaluation.

For subjective sound quality evaluation, a maximum of 50 s, including both sound source playback time and questionnaire filling time, was provided to the subject, and the time range of approximately 30 ± 5 min was adjusted in one test. Figure 4 shows the environmental conditions and the location of the loudspeaker.

Subjective sound quality evaluation was performed in the auto parts test semi-anechoic room of the Future Automotive Intelligent Electronics Core Technology Center, where background noise was maintained at less than 25 dB (A). For the playback of the sound source, a monophonic reproduction method using a loudspeaker was adopted. This is because speaker noise is low, tone changes can be expressed more clearly compared to stereo, and the reproducibility is good while being less affected by the surrounding environment [23,24]. In subjective sound quality evaluation using a loudspeaker, there is a problem in that the frequency characteristics of the reproduced sound source are distorted due to the frequency response characteristics of the speaker itself. Therefore, it is necessary to correct the frequency characteristics of loudspeakers. The correction of the frequency characteristics of the loudspeaker was performed using white noise as follows:

➀ The microphone was installed to face the speaker at a straight-line distance of approximately 1200 mm and a vertical distance of approximately 700 mm from the center of the speaker.
➁ The white noise was turned on with the loudspeaker and the noise was simultaneously measured using the microphone installed in front of the loudspeaker.
➂ The recorded white noise adjusted the sound pressure level in the third octave band by comparing it with the original frequency characteristics using an equalizer.
➃ After applying the adjusted frequency characteristics to the loudspeaker, the white noise was played/recorded.
➄ Steps 1 to 4 were repeated until the error range within ±3 dB was satisfied.

**Figure 5:**
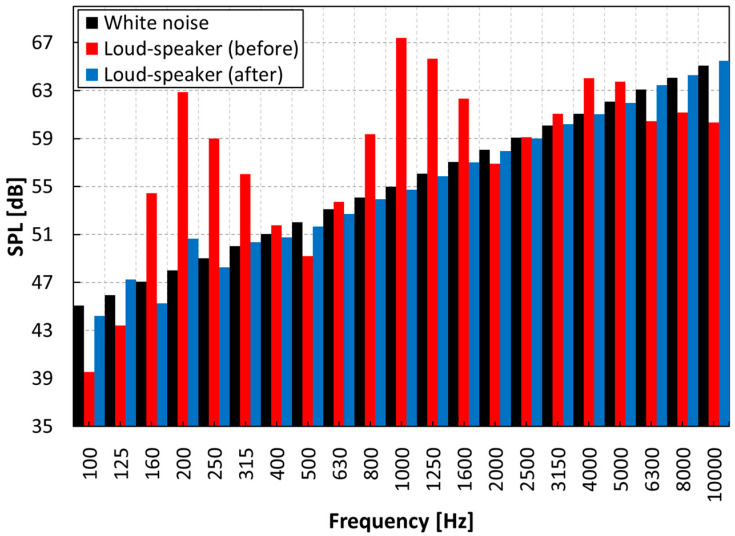
Frequency response correction of loudspeaker.

Figure 5 shows the frequency characteristics of the white noise before and after loudspeaker correction.

Forty undergraduate and graduate students at Kongju National University (35 males and five females) aged between 20 and 29 years (median age; 24.5 years) participated in the sound quality evaluation. The procedure and precautions for subjective sound quality evaluation are as follows:

➀ By conducting an evaluation targeting the general public using the product, the statistical sensibility level of the subjects using the actual product was identified, rather than the uniform arguments and perspectives of the expert group.
➁ Prior to the start of the evaluation, the subjects were trained in advance on the purpose and source of the subjective sound quality evaluation. However, by excluding product information about the sound source, the image of the brand and advertisement halo effect were suppressed.
➂ At the beginning of the evaluation, the subjects were given the initial learning process for the sound source by listening to all the sound sources to be evaluated. Through this, the subjects were able to set their own evaluation criteria.
➃ In the evaluation, the sound source was played at random such that the learning effect of the order was restricted.
➄ To prevent errors in judgment caused by exchanging opinions or information of the subjects, communication between the subjects was controlled.
➅ By controlling the time so as not to deviate from the range of approximately 30 min per subjective sound quality evaluation, errors due to the accumulation of fatigue and decreased concentration of the subject were minimized.

**Figure 6:**
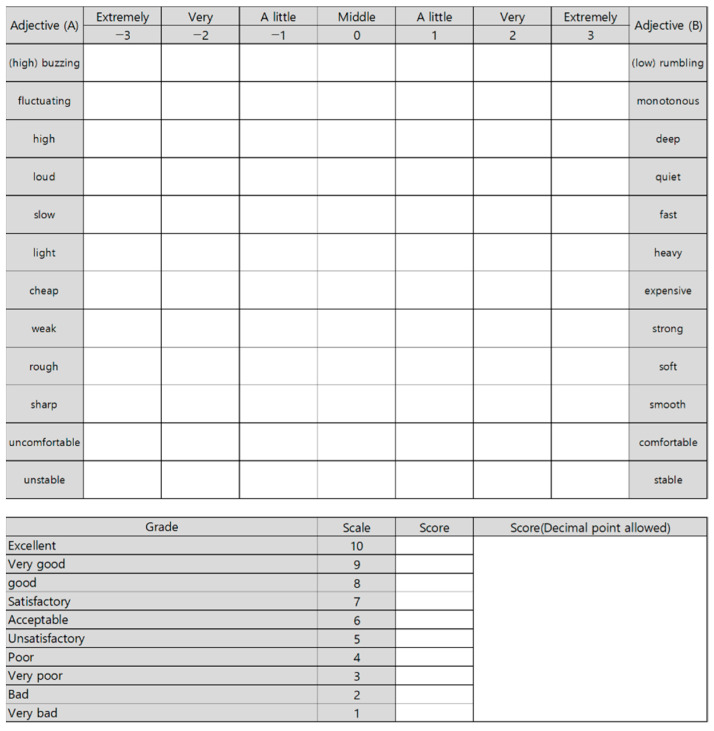
The questionnaire sheet for sound quality evaluation.

Figure 6 shows the questionnaire used for subjective sound quality evaluation. Evaluation items were set as 12 pairs of adjectives and one preference, using seven- and 10-point scales, respectively.

In subjective sound quality evaluation, the range of scores used is very different, depending on the experiences of each subject and the individual evaluation criteria. When the evaluation score was analyzed statistically, distortion occurred in the individual score range of the subjects rather than in the tendency of sound quality. Therefore, by correcting the scores of the subjects collected in the subjective sound quality evaluation through a normalization process, the difference in the range of scores between individuals can be eliminated as follows [23]:

(7) $$d_{i}=\frac{\left\{\sum_{j}^{i}(x_{\mathrm{ijk}}-\mu_{k})\right\}}{N_{s}},$$

where *d*_i_ denotes the correction value of the subject individual, i is the subject number, j is the sound source number, k is the evaluation item (adjective pairs and preference), *x* is the evaluation raw score of the subjects, *μ* is the mean of the evaluation raw score, and *N*_s_ is the number of subjects. The correction value derived from Equation (7) is corrected through the difference between the original score evaluated for each evaluation item of individual subjects, as expressed in Equation (8). The term *X′* denotes the score after correction. Thus, the average value of the individual score for each evaluation item was the same as the average of all evaluation items, thereby eliminating the difference in evaluation criteria between subjects.

(8) $${X^{\prime }}_{\mathrm{ijk}}=x_{\mathrm{ijk}}-d_{i}.$$

However, for statistical analysis, the adjusted evaluation scores must satisfy a standard normal distribution. Therefore, by performing the standardization process again as expressed in Equation (9), the evaluation score was converted into a *Z*-score, which is a standard score that satisfies the standard normal distribution. Here, *σ* denotes the mean standard deviation, and *Z* denotes the *Z*-score.

(9) $$Z_{\mathrm{ijk}}=\frac{\left({X^{\prime }}_{\mathrm{ijk}}-\mu_{k}\right)}{\sigma_{k}}.$$

We checked whether the evaluation scores post-processed through the correction and standardization processes satisfied the normal distribution through a normality test. On the basis of a significance level of 0.05, it was observed that most of the normal distributions were not satisfied owing to the presence of outliers. Therefore, outliers were extracted from the entire dataset and processed as missing values; the results are summarized in Table 3. The *Z*-score was used for outlier detection, and data outside the 95% confidence interval (±2 standard deviations) were treated as outliers. The total number of responses was 6240, and the number of missing values was 69. The final number of responses excluding missing values was confirmed to be 6171, which satisfied the normal distribution through the normality test.

#### 3.2.2. Results of Subjective Sound Quality Evaluation

**Figure 7:**
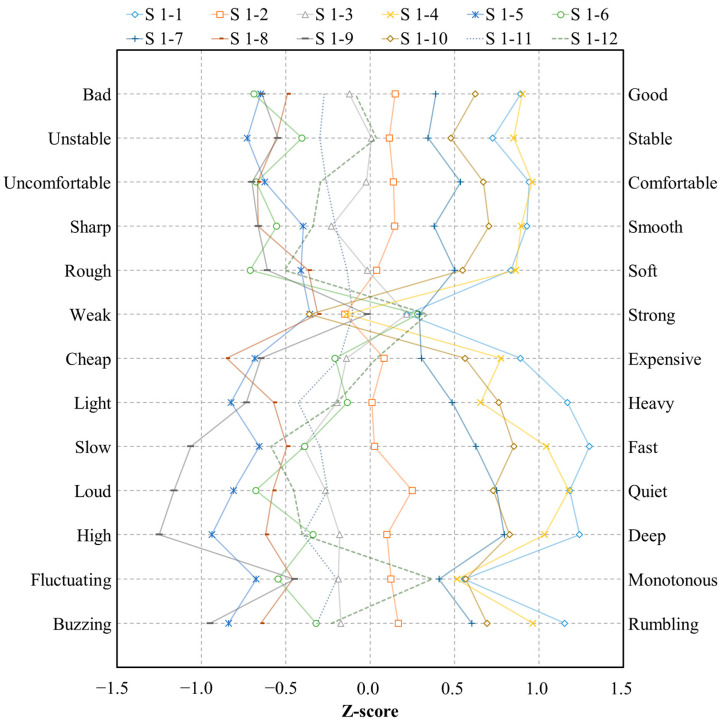
Polarity profile about subjective sound quality evaluation result.

Figure 7 shows the polarity profile obtained using the average value of the subjective sound quality evaluation results. Here, 4, 5, 6, 10, 11, and 12 represent M1 motors, whereas 1, 2, 3, 7, 8, and 9 represent M2 motors. The experimental conditions for the recorded sound source were similar to those listed in Table 1.

In the graph of the polarity profile, both the M1 and the M2 motors exhibited a generally positive response in the 0.04 N·m load condition. As the load increased, the negative score improved; thus, the negative slope characteristics were the most prominent. However, in the case of Power (strong–weak), a nonlinear characteristic without a special tendency was observed. This demonstrates that the sound quality criteria of individual subjects were very different, and it was difficult to generalize the sound quality characteristics of Power (strong–weak). Table 4 summarizes the results of the correlation analysis between subjective sound quality evaluation scores. Similarly, the coefficient of correlation between Power (strong–weak) and preference was 0.061, confirming that there was no correlation between them. In addition, the correlation coefficient between Power (strong–weak) and other adjectives was less than 0.3; therefore, it was considered ineffective.

An analysis using the polarity profile can confirm the tendency of the average sound quality; however, it has the disadvantage that it cannot confirm which factors affect the sound quality. Therefore, the causal relationship between subjective data (adjectives and preference scores) and objective data (psychoacoustic model and sound pressure level) was confirmed through multiple regression analysis. In multiple regression analysis, the sound pressure levels of the motor rotation frequency components were set as independent variables, and the regression models derived from them were referred to as the rotation frequency (RF) models. The validity of the RF model was confirmed through a comparison with psychoacoustic models. The regression estimation method of multiple regression analysis was used to select stepwise and backward elimination methods. Regression models satisfying a *p*-value of ≤0.05, and adjusted coefficient of determination (*R*^2^) of 0.6 or more were selected. The selected regression models satisfy the variance inflation factor of 3 or less and Durbin–Watson statistic of 2 ± 0.6 to verify independence between independent variables and independence between error terms. Table 5 lists the motor noise components measured in Section 2 to understand the motor rotation frequency components.

First, because Quietness (quiet–loud) means the loudness of sound, the independent variables should be related to loudness. However, the selected independent variables were *f*_E_4th_ and *f*_B_4th_, and it was difficult to consider them as factors directly related to loudness. In the case of Comfort (comfortable–uncomfortable), the selected independent variables were inversely confirmed because the factors related to the feeling of comfort were not clearly revealed. Consequently, *SPL*_Overall_ and Loudness were selected as independent variables for the RF and psychoacoustics models, respectively. Therefore, it was concluded that sound comfort was dominantly affected by loudness. The adjusted *R*^2^ values of Quietness (quiet–loud) and Comfort (comfortable–uncomfortable) are listed in Table 6: Quietness (RF model: 0.783 and psychoacoustics model: 0.842) and Comfort (RF model: 0.600 and psychoacoustics model: 0.856).

In particular, it was observed that the explanatory power of the Quietness (quiet–loud) RF model was less than that of the psychoacoustics model because the low-frequency components and overall sound pressure level were not considered. Therefore, for the sound quality factor related to the loudness of the sound, it was determined that the loudness had excellent predictive power.

Next, the adjusted *R*^2^ in Pitch (deep–high) and Smoothness (smooth–sharp) was higher in the RF model than in the psychoacoustic model: Pitch (RF model: 0.800 and psychoacoustics model: 0.748) and Smoothness (RF model: 0.714 and psychoacoustics model: 0.353). In the psychoacoustic model of Smoothness (smooth–sharp), the causal relationship with adjectives was confirmed by selecting Sharpness as an independent variable. However, the fit of the regression model was low, with an adjusted *R*^2^ of 0.353. In the RF models, *f*_E_4th_ and *f*_B_4th_ were selected as the independent variables. Because Pitch (deep–high) and Smoothness (smooth–sharp) are adjectives for pitch and sharpness of sound, respectively, the selection of high-frequency components was considered to be reasonable. In Table 7, the listed standardized coefficients were as follows: Pitch *f*_E_4th_ (1.338), Pitch *f*_B_4th_ (−0.672), Smoothness *f*_E_4th_ (1.304), and Smoothness *f*_B_4th_ (−0.696). Thus, it was confirmed that the fourth harmonic component of the electromagnetic force had a higher influence than the fourth harmonic component of the brush.

Thirdly, the adjusted *R*^2^ for Softness (soft–rough) was 0.819 in the RF model and 0.330 in the psychoacoustics model. In the RF model, *f*_M_1st_, which is a low-frequency fluctuation component due to misalignment, and *SPL*_Overall_ of the overall sound pressure level were selected as independent variables. Because Softness (soft–rough) is an adjective that indicates the roughness of a sound, the selected independent variables were considered to be reasonable. In addition, it was confirmed that the fit of the regression model was high owing to the high adjusted *R*^2^. The standardized coefficients were *SPL*_Overall_ (0.781) and *f*_M_1st_ (0.293), and the influence of the overall sound pressure level was considered to be high. In the psychoacoustic model, Roughness was selected as the independent variable. However, the fit of the regression model was low owing to the low adjusted *R*^2^.

**Figure 8:**
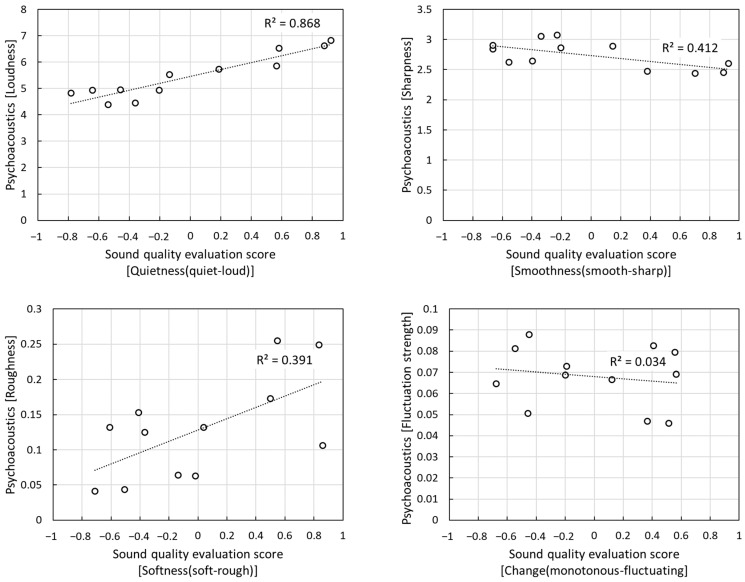
Sound quality prediction results of psychoacoustic models.

Lastly, although the psychoacoustic model had high accuracy in predicting sound quality factors related to the loudness of sound, it was considered inappropriate to apply the psychoacoustic model to other sound quality factors. Figure 8 shows the *R^2^* between the psychoacoustic model and the subjective sound quality evaluation scores. Here, Sharpness, Roughness, and Fluctuation strength, excluding Loudness, directly demonstrate the difficulty of using the model owing to its low fit.

**Figure 9:**
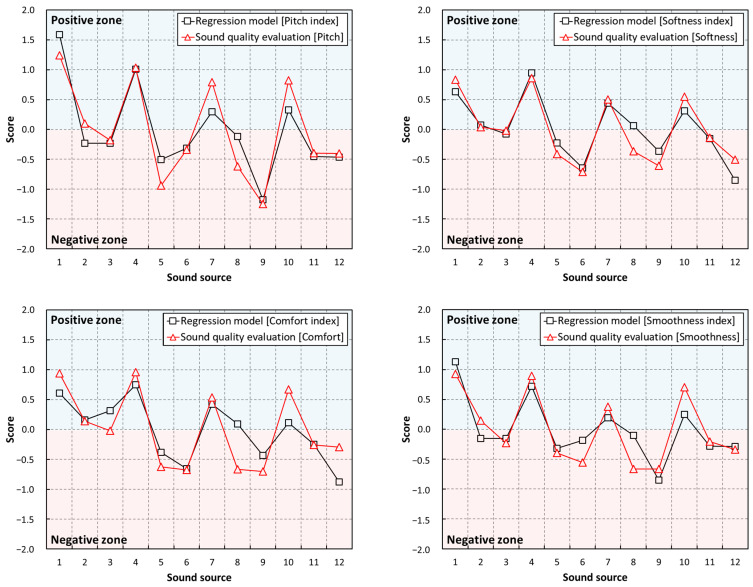
Comparison between sound quality evaluation results and regression models.

Figure 9 illustrates a comparison of the derived regression models and scores of the subjective sound quality evaluation. The subjective sound quality evaluation score was a Z-score converted through standardization that satisfies a standard normal distribution with a mean of 0 and a standard deviation of 1. Therefore, using a score of 0, it could be clearly divided into positive and negative zones. Similarly, it was possible to divide the multiple regression models into positive and negative zones. In conclusion, it was possible to derive a score of 0 as the critical sound quality value. In the graph illustrated in Figure 9, the blue and red areas indicate the positive and negative zones, respectively. The results of analyzing the sound quality using the critical sound quality value are described below.

First, in sound sources 1, 4, 7, and 10, which were common load torques of 0.04 N·m, the positive zone score was high. In sound sources 3, 6, 9, and 12, which were load torques of 0.12 N·m, negative scores were high. It was observed that, as the load torque applied to the motor increased, the sound quality was adversely affected. Next, in the clockwise rotations, the score of the sound quality components (Pitch, Softness, Comfort, and Smoothness) tended to decrease as the number of permanent magnet poles increased. Conversely, in counterclockwise rotations, the scores of the sound quality components tended to increase as the number of permanent magnet poles increased. This was predicted to be the effect of the changes in the structural and electrical resonance points according to the change in the rotational speed of the motor. Additional research is required on the noise characteristics of the motor and the effect of resonance noise owing to the dynamic characteristics.

## 4. Conclusions

In this paper, a methodology to analyze the effect of mechanical and electrical noise characteristics of small motors used in automobile interior parts on sound quality was proposed. All processes are helpful in understanding the relationship between the rotational frequency components of the motor and sound quality.

The semantic differential method using adjectives is an appropriate evaluation method to understand the multidimensional characteristics of sound quality, and the results of the sound quality analysis are highly dependent on the choice of adjectives. This suggests that appropriate adjectives for sound quality evaluation should be selected using a statistical approach.

For subjective sound quality evaluation, it is necessary to correct the score range to prevent errors in the score range between individual subjects. In addition, to ensure the reliability of statistical analysis, the standard normal distribution must be satisfied. Therefore, the data processing process of subjective evaluation scores suitable for the statistical analysis of sound quality guarantees the reliability of sound quality analysis.

The validity of the RF model presented in this study was confirmed through a comparative analysis with the psychoacoustic model. The psychoacoustic model exhibited excellent predictive power for Quietness (quiet–loud) and Comfort (comfortable–uncomfortable). However, other sound quality factors were difficult to utilize because of their low correlation. In contrast, for the RF model, the superiority of the new sound quality models was verified by exhibiting a high adjusted *R*^2^ for Comfort (comfortable–uncomfortable), Pitch (deep–high), Smoothness (smooth–sharp), and Softness (soft–rough). In addition, it was confirmed that positive (good) and negative (bad) sound qualities were determined on the basis of the critical sound quality values derived from standardized sound quality scores.

It is expected that the sound quality model and sound quality critical value can be used as basic analysis data to improve the noise performance of motors. However, further research is required to understand the effect of sound quality according to the structural resonant frequency of the motor and the change in the electromagnetic force.

## Figures and Tables

**Table 1:** Design matrix using full factorial design method.

No.	Direction of Rotation	Load Torque (N·m)	Magnet Poles	Motor Sample
1	cw	0.04	2	M2
2	cw	0.08	2	M2
3	cw	0.12	2	M2
4	cw	0.04	4	M1
5	cw	0.08	4	M1
6	cw	0.12	4	M1
7	ccw	0.04	2	M2
8	ccw	0.08	2	M2
9	ccw	0.12	2	M2
10	ccw	0.04	4	M1
11	ccw	0.08	4	M1
12	ccw	0.12	4	M1

cw: clockwise rotation; ccw: counterclockwise rotation; replicates: 3; randomized runs.

**Table 2:** Frequency analysis result for sensibility vocabulary survey.

Characteristic	Adjective Pairs	Rank (Top 15%)
Pitch	Deep	1
	High	13
Echo	(Low) rumbling	2
	(High) buzzing	5
Softness	Soft	3
	Rough	-
Speed	Slow	4
	Fast	-
Stability	Stable	6
	Unstable	-
Quietness	Quiet	7
	Loud	-
Comfort	Comfortable	8
	Uncomfortable	-
Luxury	Expensive	9
	Cheap	-
Smoothness	Smooth	10
	Sharp	-
Change	Monotonous	11
	Fluctuating	14
Weight	Light	15
	Heavy	12
Power	Weak	16
	Strong	-

**Table 3:** Information about sound quality evaluation responses.

Number of subjects (persons)		40
Questionnaire items	Adjectives (pairs)	12
	Preference	1
	Total questions	13
Amount of sound sources		12
Repeats		3
Total responses		6240
Missing values		69
Actual responses		6171

**Table 4:** Correlation analysis result of sound quality evaluation score.

	Pi-	Ec-	So-	Sp-	St-	Qu-	Co-	Lu-	Sm-	Ch-	We-	Po-	Pr-
Pi-	1.000												
Ec-	0.994	1.000											
So-	0.933	0.931	1.000										
Sp-	0.983	0.976	0.952	1.000									
St-	0.947	0.970	0.908	0.912	1.000								
Qu-	0.984	0.983	0.970	0.981	0.948	1.000							
Co-	0.962	0.973	0.977	0.955	0.971	0.981	1.000						
Lu-	0.942	0.969	0.867	0.917	0.970	0.927	0.946	1.000					
Sm-	0.951	0.964	0.967	0.968	0.941	0.974	0.985	0.943	1.000				
Ch-	0.869	0.896	0.818	0.825	0.940	0.877	0.901	0.913	0.870	1.000			
We-	0.975	0.981	0.883	0.960	0.940	0.940	0.935	0.956	0.931	0.877	1.000		
Po-	0.152	0.175	−0.054	0.022	0.211	0.051	0.073	0.253	−0.018	0.207	0.220	1.000	
Pr-	0.951	0.968	0.958	0.944	0.979	0.974	0.989	0.944	0.974	0.936	0.932	0.061	1.000

Pi-: pitch (deep–high), Ec-: echo ((low) rumbling–(high) buzzing), So-: softness (soft–rough), Sp-: speed (slow–fast), St-: stability (stable–unstable), Qu-: quietness (quiet–loud), Co-: comfort (comfortable–uncomfortable), Lu-: luxury (expensive–cheap), Sm-: smoothness (smooth–sharp), Ch-: change (monotonous–fluctuating), We-: weight (light–heavy), Po-: power (strong–weak), Pr-: preference.

**Table 5:** Rotation frequency components of motors.

Sample	Description of Source		Torque Load (N·m)					
			0.04		0.08		0.12	
			Direction of Rotation					
			cw	ccw	cw	ccw	cw	ccw
M1	Unbalance	*f*_f_ = *f*_U_	46.4 Hz	44.3 Hz	40.2 Hz	38.3 Hz	34.2 Hz	31.4 Hz
	Misalignment	*f*_M_1st_	92.9 Hz	88.7 Hz	80.4 Hz	76.7 Hz	68.3 Hz	62.8 Hz
	Brush switching and electromagnetic force	*f*_E_1st_ = *f*_B_1st_	928.6 Hz	886.9 Hz	803.6 Hz	766.5 Hz	683.2 Hz	627.6 Hz
		*f*_E_2nd_ = *f*_B_2nd_	1857.2 Hz	1773.9 Hz	1607.2 Hz	1533.1 Hz	1366.4 Hz	1255.2 Hz
		*f*_E_3rd_ = *f*_B_3rd_	2785.9 Hz	2660.8 Hz	2410.8 Hz	2299.6 Hz	2049.6 Hz	1882.9 Hz
		*f*_E_4th_ = *f*_B_4th_	3714.5 Hz	3547.8 Hz	3214.4 Hz	3066.2 Hz	2732.8 Hz	2510.5 Hz
M2	Unbalance	*f*_f_ = *f*_U_	44.8 Hz	43.2 Hz	39.0 Hz	37.0 Hz	34.1 Hz	32.6 Hz
	Misalignment	*f*_M_1st_	89.6 Hz	86.4 Hz	78.0 Hz	74.1 Hz	68.2 Hz	65.2 Hz
	Brush switching and electromagnetic force	*f*_B_1st_	448.1 Hz	431.9 Hz	390.2 Hz	370.3 Hz	341.2 Hz	326.2 Hz
		*f*_E_1st_ = *f*_B_2nd_	896.2 Hz	863.8 Hz	780.4 Hz	740.5 Hz	682.5 Hz	652.4 Hz
		*f*_B_3rd_	1344.3 Hz	1295.7 Hz	1170.7 Hz	1110.8 Hz	1023.7 Hz	978.7 Hz
		*f*_E_2nd_ = *f*_B_4th_	1792.4 Hz	1727.6 Hz	1560.9 Hz	1481.1 Hz	1364.9 Hz	1304.9 Hz
		*f*_E_3rd_ = *f*_B_6th_	2688.6 Hz	2591.4 Hz	2341.3 Hz	2221.6 Hz	2047.4 Hz	1957.3 Hz
		*f*_E_4th_ = *f*_B_8th_	3584.8 Hz	3455.2 Hz	3121.8 Hz	2962.2 Hz	2729.8 Hz	2609.8 Hz

**Table 6:** Model summary of multiple regression analysis results (rotation frequency models vs. psychoacoustic models).

Regression Model		*R*	*R* ^2^	Adj. *R*^2^	Standard Error of the Estimate	Durbin–Watson
Pitch (deep–high)	Rotation frequency model	0.914	0.836	0.800	0.362	2.443
	Loudness (psychoacoustic)	0.878	0.771	0.748	0.406	2.509
Softness (soft–rough)	Rotation frequency model	0.923	0.852	0.819	0.238	1.539
	Roughness (psychoacoustic)	0.625	0.391	0.330	0.458	1.973
Quietness (quiet–loud)	Rotation frequency model	0.907	0.823	0.783	0.373	2.539
	Loudness (psychoacoustic)	0.918	0.842	0.826	0.334	2.240
Comfort (comfortable–uncomfortable)	Rotation frequency model	0.798	0.636	0.600	0.405	1.925
	Loudness (psychoacoustic)	0.925	0.856	0.841	0.255	1.587
Smoothness (smooth–sharp)	Rotation frequency model	0.875	0.766	0.714	0.317	2.483
	Sharpness (psychoacoustic)	0.642	0.412	0.353	0.477	0.951

**Table 7:** Coefficients of multiple regression analysis results (rotation frequency models vs. psychoacoustic models).

Regression Model		Unstandardized Coefficients		Standardized Coefficients	*t*	*p*-Value
		B	Standard Error	Beta		
Pitch (deep–high)	Constant	−2.367	0.463	-		0.001
	*f*_E_4th_	0.222	0.036	1.338	6.188	0.000
	*f*_B_4th_	−0.092	0.030	−0.672	−3.107	0.013
	Constant	−4.582	0.796	-	−5.757	0.000
	Loudness (psychoacoustic)	0.837	0.144	0.878	5.807	0.000
Softness (soft–rough)	Constant	−9.167	1.368	-	−6.700	0.000
	*f*_M_1st_	0.032	0.015	0.293	2.143	0.061
	*SPL*_Overall_	0.163	0.029	0.781	5.717	0.000
	Constant	−0.621	0.279	-	−2.224	0.050
	Roughness (psychoacoustic)	4.863	1.919	0.625	2.534	0.030
Quietness (quiet–loud)	Constant	−2.391	0.477	-	−5.016	0.001
	*f*_E_4th_	0.214	0.037	1.303	5.796	0.000
	*f*_B_4th_	−0.083	0.030	−0.617	−2.744	0.023
	Constant	−4.734	0.655	-	−7.231	0.000
	Loudness (psychoacoustic)	0.865	0.119	0.918	7.297	0.000
Comfort (comfortable–uncomfortable)	Constant	−9.680	2.318	-	−4.175	0.002
	*SPL*_Overall_	0.190	0.046	0.798	4.181	0.002
	Constant	−3.800	0.499	-	−7.618	0.000
	Loudness (psychoacoustic)	0.696	0.090	0.925	7.702	0.000
Smoothness (smooth–sharp)	Constant	−1.591	0.405	-	−3.927	0.003
	*f*_E_4th_	0.159	0.031	1.304	5.050	0.001
	*f*_B_4th_	−0.070	0.026	−0.696	−2.696	0.025
	Constant	4.585	1.738	-	2.638	0.025
	Sharpness (psychoacoustic)	−1.676	0.633	−0.642	−2.646	0.024
